# The Role of Urban Growth in Resilience of Communities Under Flood Risk

**DOI:** 10.1029/2019EF001382

**Published:** 2020-03-20

**Authors:** Mona Hemmati, Bruce R. Ellingwood, Hussam N. Mahmoud

**Affiliations:** ^1^ Department of Civil and Environmental Engineering Colorado State University Fort Collins CO USA

**Keywords:** Climate Change, Flood, Resilience, Risk‐informed Decisions, Socioeconomic Development, Urbanization

## Abstract

Flood risk to urban communities is increasing significantly as a result of the integrated effects of climate change and socioeconomic development. The latter effect is one of the main drivers of rising flood risk has received less attention in comparison to climate change. Economic development and population growth are major causes of urban expansion in flood‐prone areas, and a comprehensive understanding of the impact of urban growth on flood risk is an essential ingredient of effective flood risk management. At the same time, planning for community resilience has become a national and worldwide imperative in recent years. Enhancements to community resilience require well‐integrated and enormous long‐term public and private investments. Accordingly, comprehensive urban growth plans should take rising flood risk into account to ensure future resilient communities through careful collaboration between engineers, geologists, socialists, economists, and urban planners within the framework of life‐cycle analysis. This paper highlights the importance of including urban growth in accurate future flood risk assessment and how planning for future urbanization should include measurement science‐based strategies in developing policies to achieve more resilient communities.

## Key Points

- Urban growth, resulted by socioeconomic development, plays a crucial role on rising future flood risk
- Urbanization leads to changes in hazard and exposure components of risk in flood risk assessment
- Combining urban expansion with the concept of community resilience and planning for future development helps to move towards a resilient community

## 1 Introduction

### 1.1 Problem Statement

**Figure 1:**
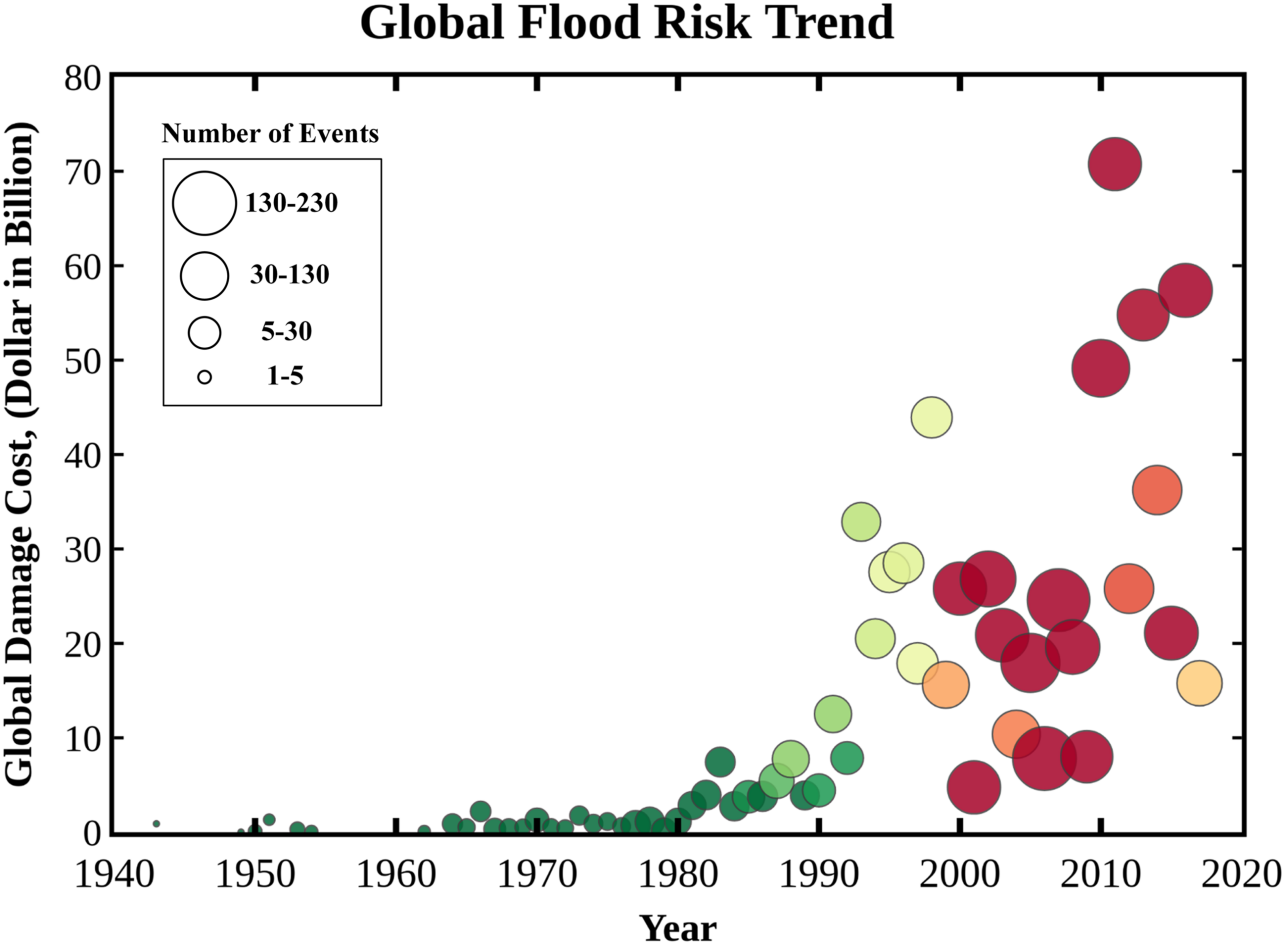
Demonstration of global flood loss trend (Munich Re, 2020). The size of bubbles represents the frequency of flood events.

Flooding is recognized worldwide as one of the costliest natural hazards (Alfieri et al., 2017; IPCC, 2014; Kreibich et al., 2017; Slater & Villarini, 2016). As illustrated in Figure 1, both frequency of and losses from global flood events have been escalating sharply, especially over the past three decades. In the United States, flooding is a major hazard to urban infrastructure; all 50 states have experienced floods or flash floods in the past 5 years (FEMA, 2019). The combination of average annual flood direct and indirect losses, reported by National Oceanic and Atmospheric Administration (NOAA), is nearly $8 billion (NOAA, 2018). In 2017, a year in which flooding from hurricanes Harvey and Irma was exceptional, flood losses to property and crop damage across the United States totaled approximately $60 billion. Moreover, the cumulative losses caused by different types of flooding events, such as riverine flooding, coastal flooding, ice jams, and hurricane‐induced flooding, among others, are higher than those associated with large‐scale natural hazards such as earthquake and tsunamis (Munich Re, 2020). Despite efforts by federal, state, and local governments to manage losses, flood hazards still threaten the lives and livelihoods of millions of people in the United States. As a result, many research and government programs are aimed at presenting improved approaches to save lives and reduce damage and economic losses.

**Figure 2:**
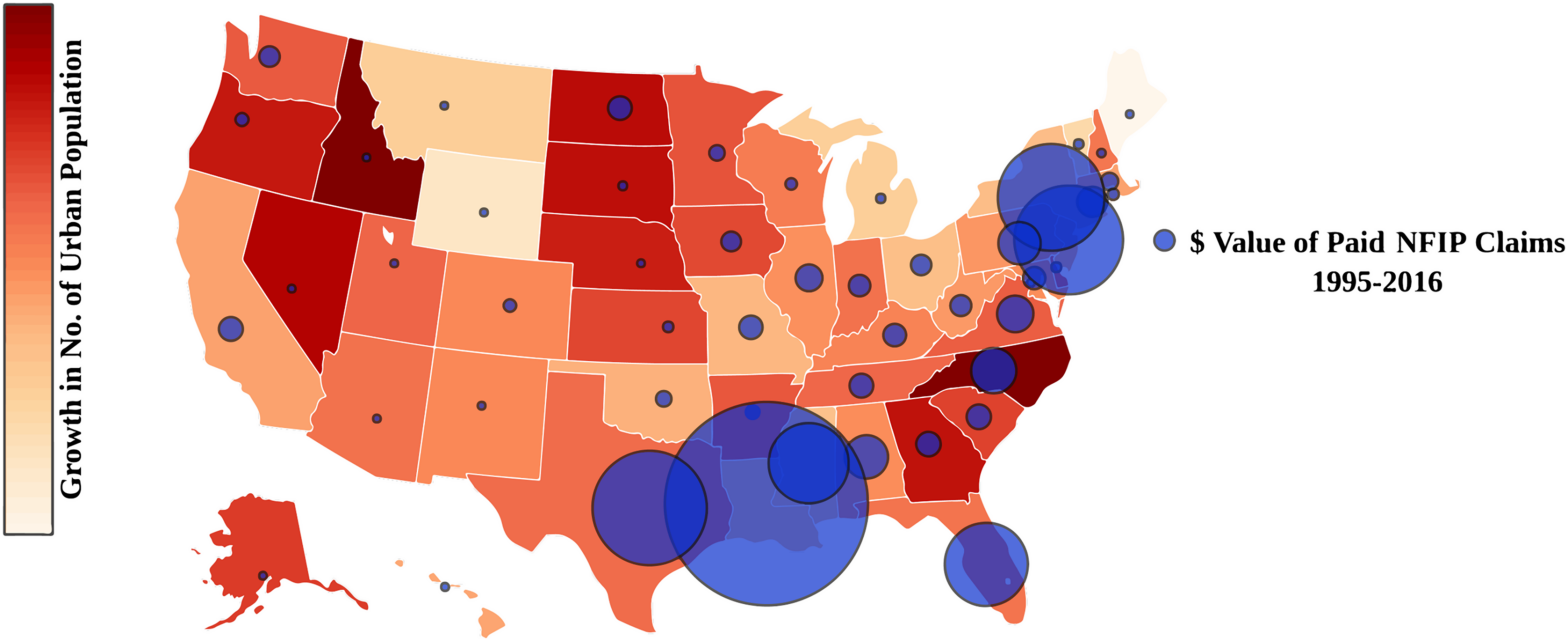
Urban population growth (U.S. Census Bureau, 2019) and value of paid NFIP claims in the United States from 1995 to 2016 (FEMA, 2019). The blue bubbles represent the proportional dollar values of claims paid by the NFIP. Colors for each state represent different percentage of urban population growth, excluding the rural areas.

Climate change and socioeconomic development in flood‐prone areas have been identified as the two fundamental factors contributing to increasing flooding losses (De Sherbinin et al., 2007; Hanson et al., 2011; Hallegatte et al., 2011; Hirabayashi et al., 2013; IPCC, 2012; Ghanbari et al., 2019; Kundzewicz et al., 2014; McPhillips et al., 2018; Moftakhari et al., 2017; Pelletier et al., 2015; Wahl & Chambers, 2016). Climate change not only plays a role in changing precipitation and flood patterns (Sharma et al., 2018) but also its combination with population and economic growth in flood‐prone areas will increase the likelihood of future severe losses in such areas. Accessibility to ports, recreational facilities, and fertile agricultural lands have made floodplains and coastal areas desirable places to live. Consequently, flood‐prone areas are experiencing steady population growth and economic development, leading to increases in flood‐related risks. As Figure 2 illustrates, many states in the United States are experiencing rapid urban population growth. The blue bubbles in this figure are proportional to the dollar values of claims paid by the National Flood Insurance Program (NFIP) for flood events from 1995 to 2016. This figure demonstrates that in almost all states both urban growth and flood hazard exist, and consequently future urban growth, coupled with climate change, can result in increase in future risk.

The encroachment of urban growth in flood‐prone areas, driven by socioeconomic development, has received less attention in comparison to climate change as a source of increasing flood risk. At the same time, in light of Presidential Policy Directive 21 (Office of the Press Secretary, 2013), planning for community resilience has become a national imperative. Therefore, comprehensive urban growth planning that reflects rising flood risk and addresses the need to enhance resilience of future communities against uncertain future severe flood events should be a key long‐term goal.

This paper summarizes key issues and challenges in planning for future urban development in flood‐prone areas from a risk‐informed perspective on community resilience. For this purpose, we strive to emphasize the significance of urban growth in rising flood risk and how engineering risk‐based approaches can be integrated with nonstructural measures in terms of socioeconomic incentives, urban planning, and land‐use policies to shape city expansion towards a resilient community.

### 1.2 Structure of Manuscript

In section 2 of this paper, we first provide some working definitions of key terms to avoid any confusion. Section 3 summarizes selected previous studies in which the effect of urban growth on flood risk, both directly and indirectly, has been considered. The studies selected are at the border between flood risk assessment components and policy implementation studies and focus on applying effective flood mitigation actions. This literature is divided into three groups, each of which is appraised based on standpoints, methods, scale of analysis, and results. The gaps are then highlighted in section 4. Finally, section 5 provides discussion and insight on how growing communities can benefit from this study and a logical path forward to create resilient communities by acknowledging the impact of urbanization and policy implementation on risk assessment.

## 2 Preliminary Definitions

### 2.1 Flood Risk Definition

**Figure 3:**
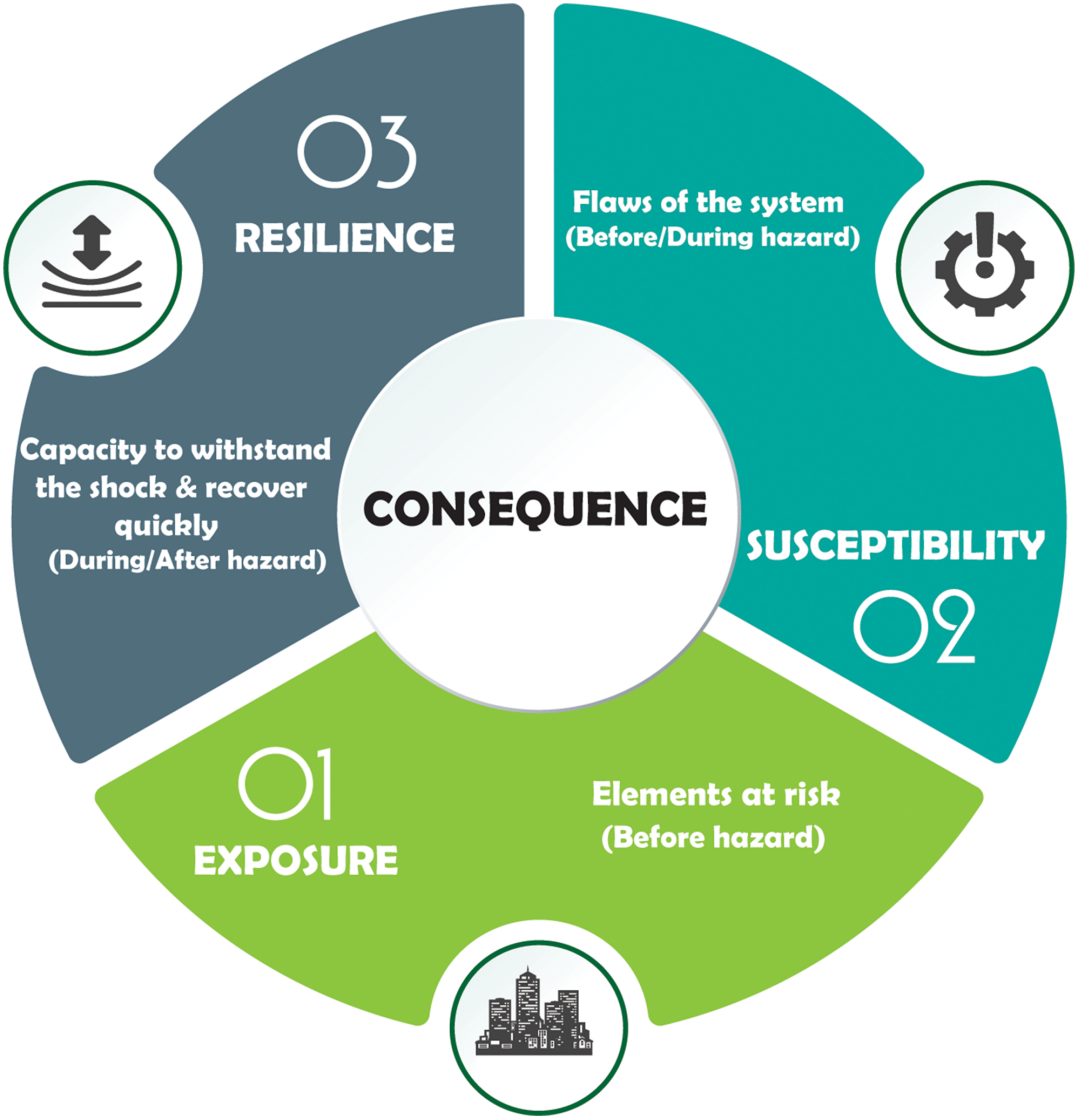
Consequence components in flood risk definition, including exposure, susceptibility, and resilience.

At the fundamental level, risk involves two components: hazard and consequences (Ellingwood, 1992). For our purpose, a hazard is an event with the potential to cause harm to people or properties. Consequence is characterized by the interaction of exposure, susceptibility (vulnerability), and resilience, as illustrated in Figure 3. Exposure is determined by the components of the built environment (i.e., people, buildings, and infrastructure) that are exposed to the hazard and can be affected by this event either directly or indirectly (Kron, 2005; Merz et al., 2010). Susceptibility is determined by flaws that make a system weak when confronted by a threat, such as flooding (FEMA 452, 2005). Resilience, on the other hand, is the capacity of the system to withstand the hazard and recover quickly (Bruneau et al., 2003). Note that exposure and susceptibility increase consequences while resilience diminishes them, implying that increasing exposure and susceptibility causes more flood loss, while improving resilience of the system reduces losses (at an additional cost of mitigation). Therefore, the notion of a consequence can be formulized conceptually by equation 1:

(1) Consequence=Exposure+Susceptibility–Resilience.

Considering the above definition of consequence, the rising flood risk phenomenon can be explained in conceptual terms. Flood risk is increasing globally due to climate change, which causes increasing frequency and intensity of extreme events (hazard), and socioeconomic development in hazard‐prone areas (exposure). Urban development, driven by population growth and the desire for increasing economic welfare, exposes more lives and assets to the risk of flooding. Therefore, planning for rational and resilient urban development not only can provide more spaces for people to live but also protect their lives and assets.

### 2.2 Role of Flood Risk on Community Resilience

Flooding frequently causes severe damage and economic losses to the built environment as well as injuries to people. Damage includes disruptions to residential neighborhoods, livelihoods, occupations and economic activities, school closures, and interrupted services from hospitals and other critical facilities. Numerous public agencies such as Federal Emergency Management Agency (FEMA), National Institute of Standards and Technology (NIST), and United Nations Office for Disaster Risk Reduction (UNDRR) and research communities are striving to improve *resilience* of communities against severe flooding by strengthening existing policies and establishing new strategies aimed at reducing flood risk through thoughtful management programs.

**Figure 4:**
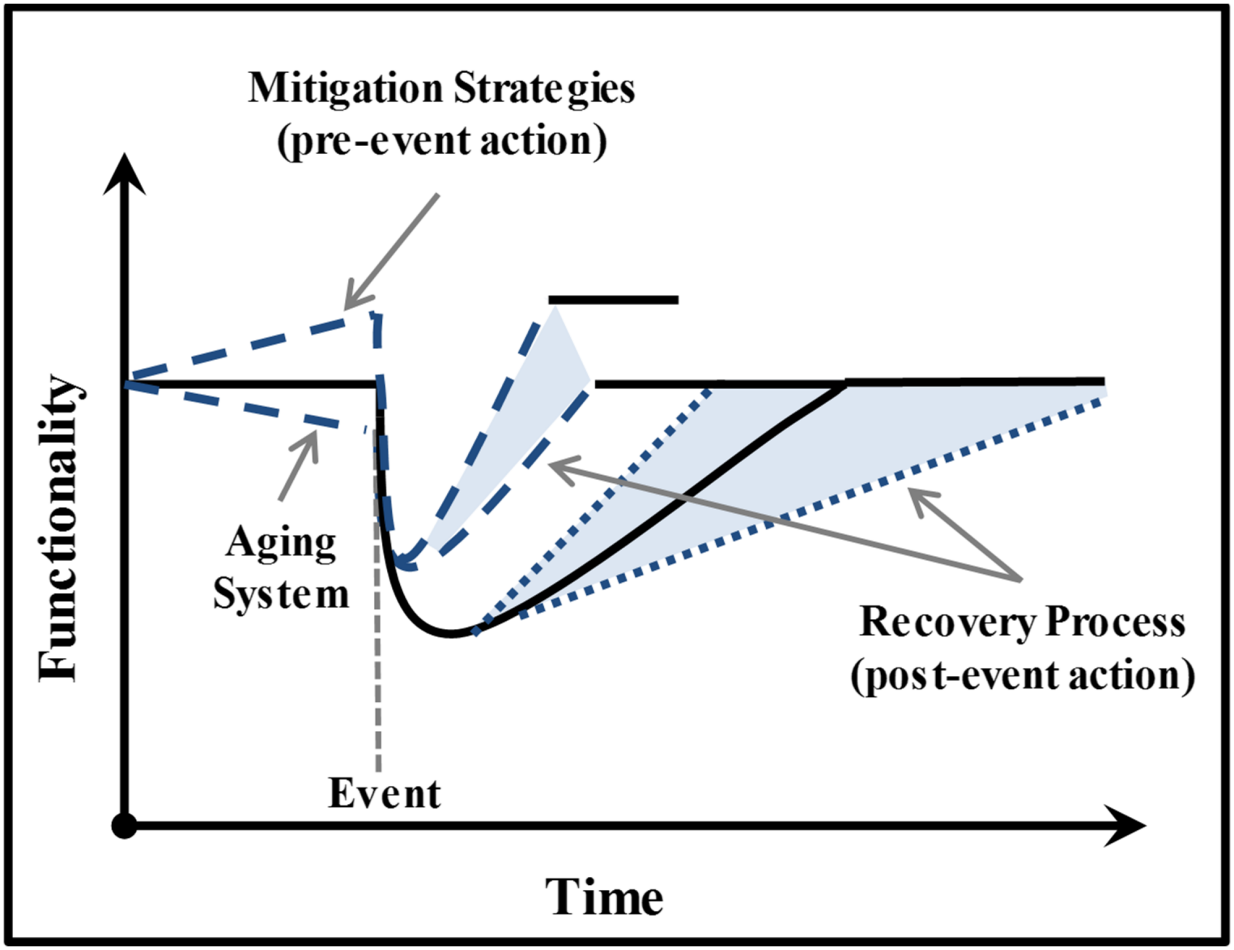
Resilience definition. (After: Bruneau et al., 2003; McDaniels et al., 2008; McAllister, 2015).

For the purposes of this review, resilience is defined as “the ability to prepare and plan for, absorb, recover from, and successfully adapt to adverse events” (NAP, 2012). Presidential Policy Directive 21 defines resilience as “the ability to prepare for and adapt to changing conditions and withstand and recover rapidly from disruptions” (Office of the Press Secretary‐PPD 21, 2013). As Figure 4 illustrates (Bruneau et al., 2003; McAllister, 2015; McDaniels et al., 2008), the resilience of an urban system is measured by its functionality of the community through time. Some of the ingredients necessary for community functionality include availability of affordable housing, services from power, water, and waste treatment, sources of employment, healthcare and educational facilities, police and fire protection, and other essential government services (Koliou et al., 2017). Rational resilience‐informed methods should be risk‐informed because of the deep uncertainties associated with climate change, the susceptibility and performance of community infrastructure, and socioeconomic support systems to severe flooding.

Two classes of actions aimed at enhancing resilience are typically taken to reduce flood consequences: pre‐event mitigation actions and post‐event recovery actions (Lin & Wang, 2016; McEwen et al., 2018). Pre‐event risk mitigation is aimed at reducing the loss in community functionality by lessening the initial consequences of the flood. Such strategies inevitably require a substantial investment in public and private funds to mitigate the consequences of a future event that is highly uncertain in magnitude and impact. post‐event recovery actions are aimed at restoring the community to a sense of normalcy within a reasonable period of time. Most communities do not have a post‐event recovery plan in place. A comprehensive urban growth management plan that aims to protect a community from rising flood risk requires having both pre‐event and post‐event plans in place.

## 3 Urbanization Impact on Future Flood Risk and Community Resilience

Traditionally, community‐level planning and land‐use policies are often focused on mitigating the impacts to flood flows from future developments. To highlight the importance of urban growth on flood risk assessment and how this appraisal can be used in form of policy implementation to create a resilient community, the literature surveyed has been selected to show the gaps and challenges. In our many years of teaching and researching this field and discussing with the well‐known specialists, we are able to narrow our literature search and illustrate the gaps and challenges with reference to the papers cited here. It is noteworthy to emphasize that the aim of this section is to draw attention to existing gaps and to provide insights for future research rather than to provide a systematic review of the current literature. Therefore, the studies mentioned in this section that sit at the research boundary of what the authors are trying to convey in this manuscript. For this purpose, we categorize the relevant literature into the following groups:

1. *Group I—Effect of urban growth on hazard assessment:* This group highlights the effect of urban growth on the hazard component of risk and how urbanization exacerbates flooding consequences. These studies mostly focus on the role of urban expansion in changes in infiltration, peak flows, and other characteristics that affect floodplains.
2. *Group II—Effect of urban growth on exposure and risk assessment:* These studies mostly focus on the flood risk assessment and the issue of rising flood risk due to climate change and socioeconomic changes. The research in this group has considered the effect of socioeconomic development instead of the actual urban expansion to account for changes in exposure component of risk. These papers often have not taken a step towards flood risk management and have not evaluated potential mitigation strategies or management plans aimed at alleviating future losses in‐depth, nor do they present a critical view on risk‐informed decision‐making to suggest comprehensive resilient urban growth plans due to changing climate and rising flood risk.
3. *Group III—Effect of urban growth on policy implementation towards a resilient community:* This group of studies addresses the role of nonstructural mitigation plans in protecting communities from flood hazards and alleviating losses. Most of the research conducted by urban planners has been aimed at identifying which mitigation plans play an effective role in alleviating flood losses during past events through qualitative approaches. They have not investigated the issue of rising flood risk quantitatively, and they have not presented effective measurement tools to control future flood losses using a comprehensive urban growth plan based on life‐cycle analysis, nor have they conducted detailed quantitative investigations of the effect of future urban growth on individual and community risk exposure.

The sections below review and compare these categories in terms of their standpoint, the methodologies they use, the scale of analysis, and results.

### 3.1 Effect of Urban Growth on Hazard Assessment

Flood characteristics, extent and depth, are not only dependent on intensity and duration of precipitation but also on topography, surface and subsurface geology, and Land‐Use and Land‐Cover (LULC). Urbanization changes the geology of the catchments and subsurface through changes in basin slopes, soil permeability, and sediment transport (O'Donnell & Thorne, 2020). It also affects the hydrology of an area through changing the amount of precipitation, adding paved areas, decreasing water bodies, reducing groundwater recharge, and reducing the capacity of urban drainage channels (Gupta, 2020). Furthermore, since urbanization transforms rural and undeveloped regions such as forests, green lands, and agricultural lands into urban areas, flooding behavior will consequently change (O'Donnell & Thorne, 2020). The complex interaction between surface and subsurface geology as well as hydrology of system and urbanization in affecting vulnerability of the built environment requires collaboration among hydrologists, geologists, and engineers to assess the changes in flood hazard. Few studies have alluded to such interaction and the associated changes in the floodplain and hydrological behavior of streams due to urban expansion (Du et al., 2012; Gori et al., 2019; Pumo et al., 2017; Suriya & Mudgal, 2012; Wijesekara et al., 2012; Zhang et al., 2018). Since the effect of urbanization on changing the floodplain behavior is a worldwide issue and can occur in any growing community, the selected studies in this section are not limited to the United States. Table S1 in the supporting information presents a summary of these papers.

#### 3.1.1 Standpoint

The main focus of this group of studies is to evaluate changes that occur in flood characteristics due to urbanization including, but not limited to, variation in peak discharge, runoff volume, time to peak, extent of the floodplain, and even rainfall patterns. For instance, Suriya and Mudgal (2012) investigated the relationship between land‐use changes and runoff responses that affect floodplains. Moreover, Zhang et al. (2018) focused on the effect of urban growth on hydrometeorology caused by Hurricane Harvey in Houston in 2017.

#### 3.1.2 Methodology

Most of the studies in this group have coupled historical urbanization patterns and land‐use projections for future scenarios to hydrological modeling software for quantifying changes in flood behavior. For instance, Du et al. (2012) coupled Cellular Automata land‐use projection models with the HEC‐HMS hydrologic model to assess the impact of urban expansion on runoff responses. Gori et al. (2019) integrated land‐use projections, obtained by a machine learning procedure, with a hydrologic‐hydraulic model that is capable of considering the site scale mitigation strategies to account for the effect of policies on floodplain extent and depth.

#### 3.1.3 Scale of Analysis

The studies in this group require high‐resolution land‐use projection and hydrologic modeling, and consequently, they mainly focus on regional and watershed extent for analysis purposes. Pumo et al. (2017) carried out an analysis for Baron Fork at Eldon river basin, located at Oklahoma, USA. Also, Wijesekara et al. (2012) focused on urbanization of the Elbow River watershed in southern Alberta, Canada.

#### 3.1.4 Results

The results of these studies can be summarized as follows:

1. Climate and land‐use changes may interact and affect the fundamental hydrological dynamics and how the processes governing basin hydrological cycle may change spatially within the community.
2. Urbanization can even have an impact on hydrometeorology, precipitation pattern, and flooding caused by hurricanes and tropical cyclones.
3. The efficiency of current site‐scale flood mitigation policies may be diminished due to urbanization in areas adjacent to a floodplain.

### 3.2 Effect of Urban Growth on Exposure and Risk Assessment

Financial and social incentives are one of the main drivers of urbanization and can have a substantial impact on the vulnerable population. Accessibility to healthcare, educational institutions, and entertainment centers are parts of these social forces that provide mental and physical motivations for people to migrate to urban areas. Moreover, from an economic standpoint, accessibility to jobs, market, and economic welfare promote people towards urban settlement. As urban expansion occurs, the population as well as public and private assets increase, which result in an increase in exposure to flood risk. Therefore, achieving a precise prediction of communities' growth over time, social and economic critical factors boosting urbanization should be considered when devising algorithms. Herein, the second category is devoted to the group of papers focusing on the role of socioeconomic developments in urban expansion and its consequence on flood risk assessment. The papers that have been selected as representatives of this group of studies include the following: Bouwer et al. (2010), Jongman et al. (2012), Hallegatte et al. (2013), Aerts et al. (2014), Güneralp et al. (2015), Muis et al. (2015), Winsemius et al. (2016), and Ward et al. (2017). Since the focus of this group is on risk assessment and not planning policies in resilience context, the selected studies are worldwide and not just specific to the United States. Table S2 in the supporting information presents a summary of these papers.

#### 3.2.1 Standpoint

Papers in this group use relatively sophisticated engineering and mathematical approaches to assess the current vulnerability of communities to flooding and to project future flood risk from an engineering and earth science standpoint. For example, in the study conducted by Jongman et al. (2012), the exposure of a community to riverine and coastal flooding was appraised in both spatial and temporal scales. Muis et al. (2015) assessed both riverine and coastal flood risk at the national scale for historic, current, and future conditions, employing a probabilistic model. The third study, led by Winsemius et al. (2016), emphasized the role of socioeconomic development and climate change on future riverine flood losses.

#### 3.2.2 Methodology

Within this group, scenario‐based analyses are often used to obtain a picture of future population statistics, and economic development in addition to climatic conditions. Since projected flood losses due to socioeconomic changes are highly uncertain, probabilistic approaches should account for the spatial and temporal uncertainties associated with these changes. On the other hand, a fully coupled risk assessment involving the flood hazard curve requires significant computational effort. Therefore, scenario‐based analyses are often adopted to simplify the risk analysis, to clarify risk for stakeholders, and to capture the spatial distribution of the flood event accurately.

In these studies, different socioeconomic scenarios have been used to project population expansion and economic growth. On the basis of these scenarios, community exposure is calculated using coupled hydrological/hydraulic models. For example, Bouwer et al. (2010) considered two different scenarios for socioeconomic development and calculated the expected losses for 42 different inundation scenarios. In the Jongman et al. study (2012), flood risk was assessed using two different methods for evaluating exposure at risk, including population and land‐use methods. The population approach used the global population and income data in order to calculate the true exposure of people and assets in hazardous areas, while the land‐use method manipulated the land‐use data to estimate exposure at risk. Muis et al. (2015) used three different models for hazard, exposure, and vulnerability. In the hazard model, 20 climate change scenarios were considered for the case of riverine flooding and three sea‐level rise scenarios were employed to calculate the inundation model for coastal flooding. In the exposure model, two inputs were required to calculate the projected exposure: urban extent and economic exposure. Projection in urban development was calculated using the economic growth and population growth under a large number of simulations to account for the uncertainties associated with population and economy growth in future projection. To calculate damage, depth‐damage functions, which estimate damage for each given inundation depth, were used.

#### 3.2.3 Scale of Analysis

Studies focusing on exposure and risk assessment often assess future flood risk at a global scale (i.e., worldwide) including the work by Jongman et al. (2012) and Winsemius et al. (2016), although there are some studies that investigated flood risk at a smaller scale (i.e., local level). For example, Bouwer et al. (2010) studied the effect of economic and population growth for a dike ring located in The Netherlands with a 740 km^2^ surface area (Lan van Heusden/de Maaskant). Muis et al. (2015), on the other hand, selected Indonesia as a somewhat larger testbed for assessing flood risk.

#### 3.2.4 Results

This group of studies has led to the following conclusions:

1. Exposure of people and assets to any type of flooding is increasing rapidly. This phenomenon is especially occurring in areas experiencing economic development and population growth. Accordingly, carefully planned urban growth is essential for controlling future losses.
2. The relative contribution of losses from climate change and socioeconomic growth is regionally dependent. In some regions, the effect of climate change is higher than socioeconomic developments while in other regions the driving force for rising losses is mainly due to socioeconomic development.
3. Urban expansion into areas that are susceptible to flooding may lead to considerable increases in risk. Therefore, thoughtful management programs and risk mitigation strategies are critical for protecting the well‐being of people and their communities. While these studies emphasized the need for adopting a holistic approach to applying nonstructural mitigation planning, only limited attempts were made to devise such an approach.
4. When considering rising flood risk, it appears that a combination of structural and nonstructural adaptive measurements, such as changes to building codes,construction of levees and barriers, and land‐use policies, can be the most cost‐effective mitigation strategies in controlling flood losses.

### 3.3 Effect of Urban Growth on Policy Implementation Towards a Resilient Community

Studies focusing on effect of urban growth on policy implementation towards a resilient community are usually aimed at determining the performance of nonstructural mitigation strategies proposed by federal, state, and local authorities to alleviate losses. Before introducing these studies, these policies adapted by local governments to control the losses are summarized. Since such management plans are directly affected by the governance system of a country, due to the familiarity of the authors to the governance system of the United States, the explained policies below as well as the selected studies in this section have focused on the United States.

Generally, mitigation strategies to reduce flood losses can be categorized into structural and nonstructural policies. Flood control actions, which are also known as structural techniques, are community‐scale projects; these include levees, dams, channel improvements, and other engineering projects that focus on flooding itself rather than on the impact on people. These policies are instituted to control the extent of flooding within the community (Burby & French, 1981). Based on a report from the Army Corps of Engineers (USACOE, 2002), flood monetary losses from 1991 to 2000 were about US$45 billion but could had been an additional US$208 billion had flood protection structures not been available. Despite the benefits of these mitigation plans, they have some drawbacks as well. Most significant, they create a path to more development in susceptible flooding zones by creating a safe feeling. Consequently, if the intensity of the flood event is higher than the capacity of the protection structure, which is quite likely due to a changing climate and the occurrence of more extreme events, the community might incur catastrophic losses for which it is unprepared. Another issue of these techniques is the cost of investing in flood protection structures. For instance, between 1940 and 2000, over $100 billion has been spent in the United States in constructing such structures (Burby & French, 1981).

Nonstructural measures, on the other hand, are public‐sector flood mitigation initiatives that are intended to change peoples' behavior to keep them out of the floodplain and reduce the *exposure* to hazard rather than the *hazard* itself. These measures include floodplain maps to inform the community about the hazardous areas, regulation of development to require the use of flood‐resistant design, and regulation of construction technique through various actions. These actions include the following: building regulation (elevation requirement, zoning, wetland protection regulation, critical areas destination, density exchange, and cluster), capital improvement policies, land acquisition policies, socioeconomic incentives (taxation), and public awareness programs. These policies are mostly effective for the communities experiencing urban expansion since there is an opportunity to implement as the community expands.

In the United States, the community resilience concept in flood hazards has been implemented though some *qualitative* programs by FEMA such as NFIP and Community Rating System (CRS) (FEMA, 2017a). Based on the NFIP program, communities that are inside a 100‐year floodplain are required to purchase flood insurance to recover from flooding events. On the other hand, the CRS, a voluntary program established by FEMA in 1990, provides incentives for communities that are inside the 100‐year FEMA floodplain to adopt flood mitigation strategies and to benefit from reduced premium rates (FEMA, 2017b). The comprehensive assessment conducted by Sadiq et al. (2020) provides a state‐of‐the‐art review of the effectiveness of the CRS and other policies adopted by FEMA to control flooding consequences in the United States.

In the next section, references that examine the effect of policy implementation on flood loss are evaluated. Using the same approach as in the previous section, the studies in this group are classified based on their standpoints, methodologies, scale of analysis, and results. The representative papers that will be discussed in this section are as follows: Birkland et al. (2003), Brody et al. (2007), Glavovic et al. (2010), Brody et al. (2011), Highfield et al. (2012), Berke et al. (2014), Brody et al. (2014), Sadiq and Noonan (2015). The main features of these publications are also summarized in Table S3 in the supporting information.

#### 3.3.1 Standpoint

These papers assess the effect of nonstructural measures on property damage, along with the most effective practices for minimizing losses. Emphasis is placed on identifying the management policies that have been responsible for increasing flood losses and the consequences of characteristics, patterns, and attributes corresponding to the built environment, which plays a role in flood risk. Other studies in this research group concerned about resilience and recovery of communities and assess their preparedness in flooding events (Berke et al., 2014). They do not apply any engineering and risk‐based approaches for their purpose and usually rely on the survey distribution and regression analysis based on collected data from past events. Some other studies in this group focus on resilience practices using CRS guidelines and evaluate the effectiveness of the policy implementation on reducing the consequences of flooding hazards (Sadiq & Noonan, 2015).

#### 3.3.2 Methodology

Two different methodologies are commonly used to analyze data in these manuscripts, both of which are empirical in nature:

1. Regression analysis: Descriptive statistics from insurance claims or other quantitative reports from past events have been used, considering specific spatial and temporal scales. The dependency of flood loss (dependent variable) on independent variables, such as high‐density/low‐density development and household income, is evaluated using regression analysis.
2. Survey distribution: This type of analysis is mainly applied to studies concerned with the effectiveness of enforced policies and management programs on flood loss. Comprehensive surveys are distributed among local, state, and federal authorities to evaluate how the policies have been employed and to identify the corresponding consequences.

For regression‐based studies, the assessed variables are as below:

1. Dependent variable: flood loss, property damage, and high flood damage event.
2. Independent (control) variables: high‐density development, low‐density development.
3. Planning control variables: impervious surface, soil permeability, floodplain area, availability of structural measurements like dams, wetland alteration, precipitation, and storm surge.
4. Baseline socioeconomic variables: adjacent damage, housing value density, number of housing units, and median household income.
5. Natural environment variables: precipitation, floodplain area, stream density, and flood duration. For survey‐based studies, the variables are those that had been adapted to communities as nonstructural mitigation policies such as the FEMA CRS rating system (2017a).

#### 3.3.3 Scale of Analysis

The analysis for such studies is usually at the local or state level and is spatially dependent on area, since policy development, implementation, and enforcement usually is conducted by local governments. For example, Brody et al. (2007) considered 383 nonhurricane flood events across 54 coastal counties in Florida from 1997 to 2001. On the other hand, studies of the effectiveness of land‐use policies in controlling flooding events usually are conducted on the national level. A typical example is a study by Sadiq and Noonan (2015), which examined the effectiveness of such policies on national scale.

#### 3.3.4 Results

The following conclusions are common to most of the papers in this group:

1. Development pattern effect: High‐density development reduces the dollar amount of insured flood losses, while low‐density urban development patterns increase the losses.
2. Geophysical and hydrological variables: Storm surge and extreme precipitation are responsible for flood losses. Jurisdictions in which a relatively high percentage of properties are located in a100‐year floodplain experience higher losses in flood events. Furthermore, wetlands play an effective role in reducing flood losses, and opening wetlands to construction increases runoff and flood damage. Several studies have suggested utilizing wetlands as a natural mitigation plan for decreasing flood effects on the built environment. In contrast, dams may not alleviate flood losses significantly if planning strategies do not consider the influence of biophysical, socioeconomic, and planning decision variables.
3. Socioeconomic variables: Increases in the number of housing units in flood‐prone areas will increase the losses in the case of flooding events.
4. Management policy effects: The effectiveness of a comprehensive program such as NFIP depends highly on community characteristics. Moreover, the studies have shown that land‐use policies are not very effective for existing communities. For such communities, a combination of structural and nonstructural actions should be adopted to protect the community from flooding hazards. Moreover, current land‐use strategies may not be sufficiently effective to protect the natural areas from flooding. Such policies may not be capable of prohibiting construction in floodplains. Therefore, the federal government should intertwine with the local plans and halt development in these areas.

## 4 Research Issues and Challenges

Using the above literature review, the following gaps and challenges can be highlighted:

1. According to a more recent Group I study (Gori et al., 2019), coupling hydrologic/hydraulic models with urban growth models will result in large uncertainties both in terms of land‐use projections and hydrodynamic models.
2. Group I studies have not evaluated flood risk and its variation considering urban expansion. Moreover, the lack of intertwined effect of urbanization and policy implementation in hazard assessment is obvious.
3. There is a lack of collaborative work between geologists, hydrologists, social scientists, economists, and engineers to fully evaluate the effect of urbanization on future flood risk. This collaboration is bound to improve planning regulations, building codes, society participation in voluntary flood mitigation measures through creating some socioeconomic incentives such as tax returns and decreasing the flood insurance premiums (O'Donnell & Thorne, 2020).
4. Group II studies have mostly evaluated the effect of socioeconomic development by considering different scenarios for population and economic growth to account for changes in exposure component of risk. Although urban growth is a direct result of socioeconomic development, the spatial distribution of the built environment, that is, buildings and infrastructure, resulted by urban growth can have an extensive effect on the losses. Current studies have seldom paid attention to this point in their proposed frameworks.
5. The main concern of Group II studies is risk assessment under current conditions and future projections. The nonstationary effects of future flood risk drivers—population and economic growth as well as changing climate—have been considered by utilizing scenario‐based analysis. Although this approach can be used to predict future conditions, more sophisticated stochastic approaches are required to acknowledge the dynamic impact of urban growth regarding social and economic incentives boosting urbanization and to account for uncertainties associated with this nonstationary in the process of flood risk assessment.
6. Group II studies have not considered a comprehensive role of policy implementation as mitigation strategies and adaptive policies in evaluating future flood risk. Although some studies have considered limited mitigation strategies in their risk assessment framework, mainly structural measures such as building elevation, they have not considered the effect of nonstructural measures such as land‐use planning and socioeconomic stimuli adopted by these programs on future flood losses. Consequently, they have not offered any practical solutions for moving towards a resilient community, considering urban growth.
7. Group III studies generally have focused on nonstructural mitigation plans and their effectiveness in practice, as noted in the review by Tyler et al. (2019). Such studies rely heavily on empirical approaches and statistical data collected from surveys or insurance claims to determine the effectiveness of land‐use policies. Because the models developed are incident‐specific, they share the deficiency of all regression‐based models—their perspectives are backward rather than forward—and their extrapolation to other flood events is questionable. They lack the science or engineering perspective needed to evaluate the effectiveness of an adopted policy on future projections. Moreover, most of these models do not consider the effect of either climate change or future socioeconomic changes on the effectiveness of such land‐use programs.
8. In all three groups, the lack of resilience and recovery‐based plans is notable.
9. Currently, there are two methodologies to study the effect of adaptation measures on a regional and local scale: the predictive top‐down approach and the resilience bottom‐up approach. (Carter et al., 2007; Dessai & van der Sluijs, 2008; Kwadijk et al., 2010). The top‐down approach is used when different climate change and socioeconomic adaptation measures are applied to assess the impact and consider the uncertainties associated with the flood risk assessments. At the moment, few studies that address the issue of urban growth considering the future flood risk take a predictive top‐down management approach (Muis et al., 2015) instead of a resilience‐based bottom‐up approach. A limitation with these approaches is that they are not powerful enough in terms of their applicability in flood risk management since the decision‐makers need to have more accurate information to adopt proper management plans. In addition, these approaches do not take differences in characteristics of communities into account. Studies have shown that bottom‐up approaches are more accurate in decision‐making when it comes to assessing mitigation strategies and identifying near‐optimal plans. In other words, examining the adaptive capacity and adaptation measures required to improve the resilience and robustness of a community exposed to socioeconomic development need to be captured by bottom‐up methods that focus on vulnerability and risk management. (Kwadijk et al., 2010)
10. Resilience and recovery concepts from an engineering risk‐informed decision‐making perspective have not been considered in these studies. Although Group III studies have investigated some mitigation and management policies, to support measures such as those embedded in the CRS program, the policies are qualitative and often rely on personal judgment. Therefore, there is an essential need for developing quantitative risk‐informed community resilience and recovery frameworks that are measurement science‐based and that take future urbanization into account.
11. Finally, there is a lack of a risk‐informed, life‐cycle perspective in the categories mentioned. Both urban growth and process of policy implementation for community resilience have long life cycles; that is, they develop slowly over decades. None of the studies reviewed appears to have recognized the role that life‐cycle analysis should play in developing long‐term strategies for mitigating future flood risks. Although Group II studies have assessed the risk through time and predicted an increasing trend in flood risk due to its drivers—climate change and socioeconomic changes—they have not used any life‐cycle engineering analysis to attempt to identify optimal strategies to mitigate risk. Moreover, Group III studies have not accounted for the effectiveness of the policies through time while considering the rising cumulative losses of flood events.

## 5 Discussion and the Path Forward

With urbanization on the rise worldwide, the interaction between hazard, exposure, and impact is ever‐evolving. As such, continuous updating of mitigation and recovery policies aimed at minimizing risk and making communities more resilient is needed. A key to achieving this is to recognize the necessity for developing new frameworks, which are currently nonexistent, that account for the role of urbanization when assessing future flood vulnerabilities and risks. The inclusion of such role will allow policymakers the opportunity to explore various alternative policies that are both socially and economically acceptable not only for current but also for future generations.

**Figure 5:**
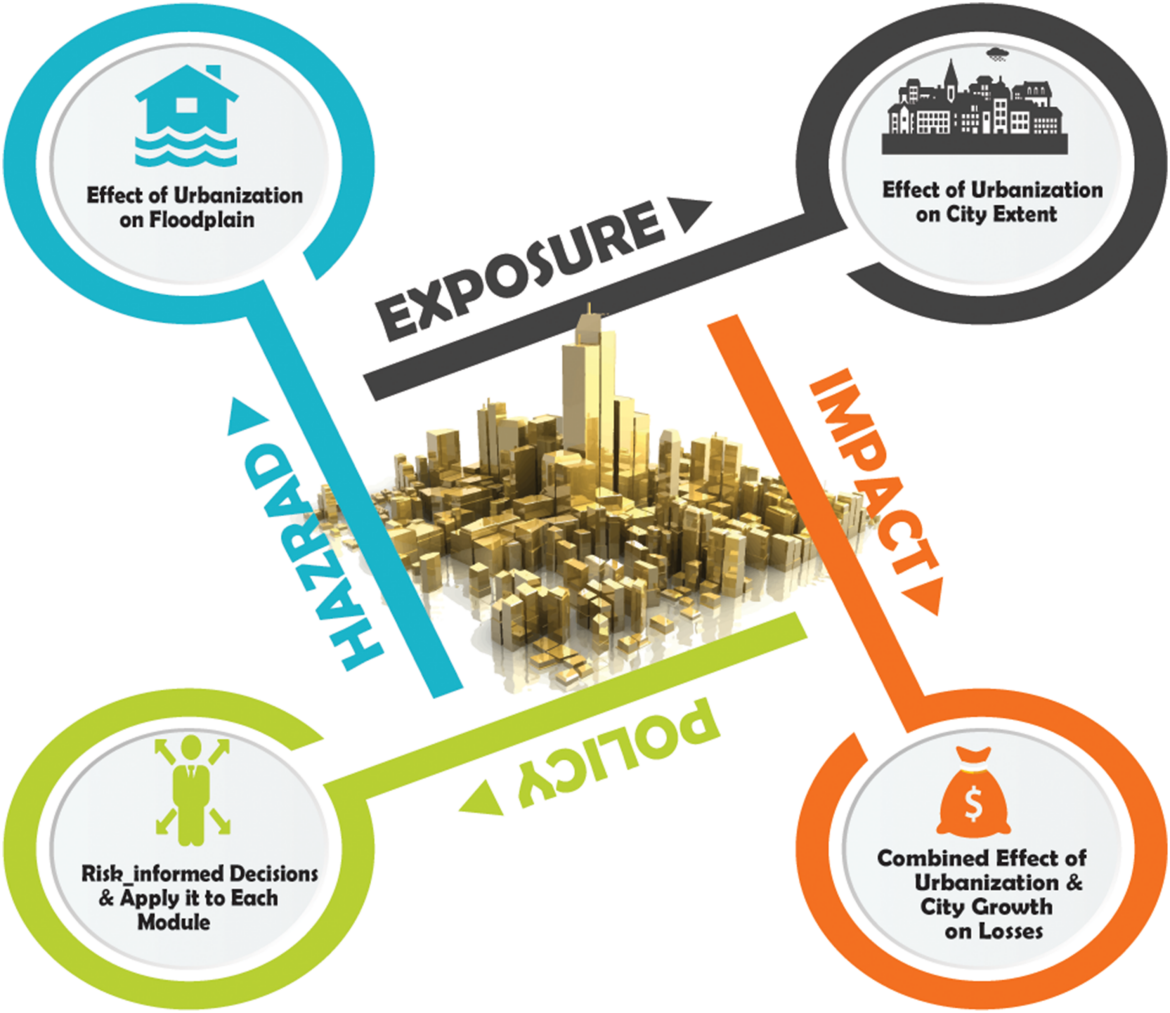
Proposed framework for a comprehensive risk‐informed planning of future urban growth. The policy module is added to the conventional framework for risk management. Submodules should be refined for considering the effect of urban growth and mitigation policies.

The path forward for future advances should include the following:

1. As mentioned before, flood risk consists of two components—hazard and consequence. When evaluating flood risk, researchers have relied on inundation maps to characterize the hazard footprint. Since urbanization changes the topology, geology, and hydrology behavior of the region, a more holistic and continuously updated framework should be embraced to provide a better prediction of the floodplains. Moreover, exposure should be assessed by considering the socioeconomic changes in the community as influenced by different incentives. Traditionally, quantifying risk has been realized in previous studies by combining the hazard and exposure. We propose that another module—“policy implementation”—be added to the conventional risk assessment framework aimed at achieving a fully risk‐informed decision‐making approach. The Disaster Risk Reduction (e.g., Bubeck et al., 2017) measures not only require realistic prediction of future risk but also the effective implementation of policies within a life‐cycle engineering context. Therefore, to assist communities and officials from gaining benefits from flood risk assessment research, adding the “policy implementation” step in flood risk assessment framework will provide an opportunity to plan for and build communities that are resilient against flood events. In addition, as illustrated in Figure 5, proper risk‐informed decision making at the community level requires hazard and exposure modules that permit different policies to be tested and provide inundation and exposure maps. Using such information will aid officials to assess the effectiveness of flood risk mitigation policies.
2. To model the process of urban growth, behavioral aspects, such as the decisions of city governments, residents of the community, and the stakeholders such as construction companies who actually gain monetary benefit from the growth should be considered.
3. Urban growth should be modeled so as to account for community resilience objectives in the growth process, such as physical, economic, social, and governance functionalities. Correspondingly, multiobjective optimization is needed to achieve the desired performance level among these competing objectives by adopting different nonstructural policies, including taxation, acquisition, land‐use planning, and zoning.
4. To enable resilient communities, research should adopt an integrated risk‐based engineering and urban planning framework. To propose effective mitigation and management policies, first of all, risk needs to be accurately quantified by accounting for the role of urbanization as influenced by existing policies. Then, based on the methodology presented in section 2, resilience objectives can be measured and the effect of different mitigation strategies on these objectives can be quantified.
5. Public and private organizations still make decisions based mainly on the initial costs and do not consider a life‐cycle approach, despite the potential significant savings in the long run. Regardless of the approach used in decision making, investments in flood risk mitigation must be made for the long term. Cumulative losses from flooding events could ultimately trigger social and economic instabilities such as population dislocation. Therefore, there is an essential need for research that combines engineering approaches in risk assessment with nonstructural mitigation policies through time. Such an approach will better inform community decision‐makers about mitigation strategies that will move a community towards heightened resilience to flood events.

## 6 Conclusions

Socioeconomic development, driven by economy and population growth, and climate change are recognized as drivers for rising future flood risk. The encroachment of urban growth in flood‐prone areas, which is a direct result of socioeconomic development, has received less attention in the literature. This paper summarized the key issues considering the future urban development in flood‐prone areas from a risk‐informed community resilience stance.

We categorized carefully selected literature that could be a representative for related research in the field into three groups, including studies focusing on *Effect of urban growth on hazard assessment*, *Effect of urban growth on exposure and risk assessment*, and *Effect of urban growth on policy implementation towards a resilient community*. We reviewed and compared these studies in terms of their standpoint, the methodologies they used, scale of analysis, and results. Gaps in the literature were identified in the context of risk‐informed decision‐making directed towards making future cities more resilient to flooding. Significant gaps include the following:

1. The lack of measurement science‐based approaches to assess the effect of urbanization on flood risk assessment.
2. The lack of risk‐informed planning approaches at the government level that enable the concept of resilience to be integrated into public planning and policy decision regarding future community development.
3. The lack of flood mitigation strategies based on life‐cycle analysis that is constraint by nonstructural mitigation measures.

The critical appraisal of this literature can inform future research that examines tradeoffs between costs and benefits of future land development regarding uncertainties in flood hazard, performance of the built environment, and population and economic growth during the remainder of the 21st century.

## Supporting information



Supporting Information S1

Table S1

Table S2

Table S3
