# Supplementary material for: The Role of Urban Growth in Resilience of Communities Under Flood Risk
Source: Earths Future. 2020 Mar 20;8(3):e2019EF001382. doi: 10.1029/2019EF001382 (PMC7375139; doi:10.1029/2019EF001382)
Supplement: Supplementary file 1 — Supporting Information S1 [file EFT2-8-e2019EF001382-s001.docx]

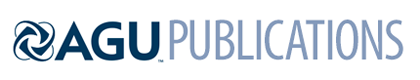


*Earth’s Future*

Supporting Information for

**The Role of Urban Growth in Resilience of Communities under Flood Risk**

Mona Hemmati^1^, Bruce R. Ellingwood^1^, and Hussam N. Mahmoud^1^

^1^Department of Civil and Environmental Engineering, Colorado State University, Fort Collins, CO, USA

**Contents of this file**

Tables S1 to S3

**Additional Supporting Information (Files uploaded separately)**

Captions for Tables S1 to S3

**Introduction**

Tables S1 to S3 provide detailed information for the selected studies focusing on *Effect of urban growth on hazard assessment*, *Effect of urban growth on exposure and risk assessment*, and *Effect of urban growth on policy implementation towards a resilient community*, respectively. These studies sit at the research boundary of what the authors are trying to convey in this manuscript. The provided tables review and compare these studies in terms of their standpoint, the methodologies they utilize, scale of analysis, results, and flood types.

Table S1. Summary of Studies Focusing on the Effect of Urban Growth on Hazard Assessment

Table S2. Summary of Studies Focusing on the Effect of Urban Growth on Exposure and Risk Assessment

Table S3. Summary of Studies Focusing on the Effect of Urban Growth on Policy Implementation towards a Resilient Community
