# Supplementary material for: The Role of Urban Growth in Resilience of Communities Under Flood Risk
Source: Earths Future. 2020 Mar 20;8(3):e2019EF001382. doi: 10.1029/2019EF001382 (PMC7375139; doi:10.1029/2019EF001382)
Supplement: Supplementary file 2 — Table S1 [file EFT2-8-e2019EF001382-s002.docx]

Table 1
Summary of Studies Focusing on the Effect of Urban Growth on Hazard Assessment

| Authors | Purpose of the Study  (Standpoint) | Method | | Scale of analysis | | Key Results | Flooding type |
| --- | --- | --- | --- | --- | --- | --- | --- |
| Suriya and Mudgal^d^ | Evaluating changes in 100-year floodplain resulted by urbanization in the watershed. | | Remote sensing and GIS,  HEC-HMS, HEC-RAS | | **Spatial Scale:**  Thirusoolam watershed, Palar basin, India  **Temporal Scale:**    1976-2005 | - Floodplain extend increased for the period of 1976-2005 due to urban expansion.  - Depth of water in the floodplain increased for the period of 1976-2005 due to urban expansion.  - Flood management should be based on the boundaries of watershed, not on administrative areas.  -Planning for urban expansion should take into account the consequences of changes in floodplain due to urbanization. | Riverine Flooding |
| Du et al.^a^ | Assessing the effects of urbanization on annual runoff and flood events. | | Coupled HEC-HMS and (CA-Markov) | | **Spatial Scale:**  Qinhuai River basin, China  **Temporal Scale:**  1988-2018 | - Slight increases in mean annual runoff of the whole watershed as a response to urbanization.  - Potential changes in peak discharge and flood volume with increasing impervious surface showed linear relationships  - Daily flood peaks flow and flood volumes increase with imperviousness for all flood events | Riverine Flooding |
| Wijesekara et al.^e^ | Assessing the impact of future land-use changes on hydrological processes. | | Combined  CA/MIKE-SHE | | **Spatial Scale:**  Elbow River watershed, Canada  **Temporal Scale:**  2001–2031 | - Urbanization increases overland flow and reduces total water supply via the Elbow River  - A potential significant negative impact on the sustainability of ground/surface water supplies and groundwater storages in the future in the watershed in addition to an increased risk of flashy floods. | Riverine Flooding |
| Pumo et al.^c^ | Evaluating the effect of urbanization on watershed hydrology. | | A Physics-based hydrologic model and CA | | **Spatial Scale:**  Baron Fork at Eldon river basin-USA  **Temporal Scale:**  Current to 2080 | - Climate and land use changes may interact and affect the fundamental hydrological dynamics  - The processes governing basin hydrological response may change with spatial scale with changes in land uses. | Riverine Flooding |
| Zhang et al.^f^ | Assessing urbanization effects on rainfall and flooding | | The Weather Research and Forecast | | **Spatial Scale:**  Houston, USA  **Temporal Scale:**  1950-2017 | - Probability of flood events across the studied basins increased on average by about 21times due to urbanization.  - The effect of urbanization on storm-induced extreme precipitation and flooding should be more explicitly included in global climate models.  -Urbanization needs to be taken in to account in assessing the flood risk for highly urbanized area. | Storm-induced extreme precipitation |
| Gori et al.^b^ | Characterizing urbanization impacts on floodplain. | | A coupled hydrologic, hydraulic, and machine learning | | **Spatial Scale:**  Cypress Creek watershed, USA  **Temporal Scale:**  2011 - 2050 | - 100-year floodplain can expand by up to 12.5% across the watershed as a result of projected development in 2050 using current stormwater mitigation policies.  - Incremental land use changes can significantly alter the reality of flood risk.  - Existing land use policies may be insufficient to mitigate impacts from future development. | Riverine Flooding |

^a^Du et al. (2012), ^b^Gori et al. (2019), ^c^Pumo et al. (2017), ^d^Suriya and Mudgal (2012), ^e^Wijesekara et al. (2012), ^f^Zhang et al. (2018).
