# Supplementary material for: The Role of Urban Growth in Resilience of Communities Under Flood Risk
Source: Earths Future. 2020 Mar 20;8(3):e2019EF001382. doi: 10.1029/2019EF001382 (PMC7375139; doi:10.1029/2019EF001382)
Supplement: Supplementary file 3 — Table S2 [file EFT2-8-e2019EF001382-s003.docx]

Table 2
Summary of Studies Focusing on the Effect of Urban Growth on Exposure and Risk Assessment

| Authors | Purpose of the Study  (Standpoint) | Method | | Scale of analysis | | Key Results | Flooding type |
| --- | --- | --- | --- | --- | --- | --- | --- |
| Bouwer et al.^b^ | Evaluating the effect of socio-economic development and climate change on future river flood losses. | | Scenario-based  analysis | | **Spatial Scale:**  Dike ring area 36 located in Netherland with a 740${Km}^{2}$ surface area  **Temporal Scale:**  1970-2040 | - Due to socio-economic changes the Annual Expected Loss (AEL) increased from 25% to 172% for the year 2000 to 2040, respectively. For climate change this variation was 46% to 201%. Considering these two factors simultaneously, the AEL increased from 96% to 716%.  - Growing exposure due to socio-economic change was exacerbated by climate change leading to an additional increase in expected loss.  - The urban expansion and the geographical location of communities into areas susceptible to flooding resulted in a considerable increase in risk. | Riverine Flooding |
| Jongman et al.^d^ | Calculating flood exposure due to riverine and coastal flooding. | | Land-use method and population method | | **Spatial Scale:**  Global  **Temporal Scale:**  1970-2050 | - Up to 2010, the highest number of people and asset at flood risk were in the Asian and developed countries, respectively.  - Exposure to coastal and fluvial flooding increased for 1970-2010 and projected for 2010-2050.  - The results confirmed that the steering exposure occurred in areas experiencing economic development and population growth. | Riverine and Coastal Flooding |
| Hallegatte et al.^f^ | Quantifying present and future coastal flood losses while accounting for existing and future flood defense. | | Scenario-based analysis | | **Spatial Scale:**  Global (136 cities)  **Temporal Scale:**  Four-time steps:  2005- 2030 - 2050-2070. | - Current standards for protecting cities are not enough for defending coastal cities from future flood risk.  - Probability of flooding needs to be reduced to keep the flood losses at the same level as current situation.  - Modifying the current codes can reduce flood events but still the magnitude of loss will increase due to flood intensity. This point leads to deficiencies associated with structural measurements. | Coastal Flooding |
| Aerts et al.^a^ | Assessing flood resilience strategies for controlling the flood risk of coastal cities | | Scenario-based analysis | | **Spatial Scale:**  A megacity scale (New York)  **Temporal Scale:**  Current to 2080 | - By considering rising flooding risk, it seems a combination of structural and non-structural adaptive measurements such as building codes and levee and barriers can be the most cost-effective mitigation strategies in controlling the flood losses for the case study.  - The source of investment on the city mitigation plans can come from household, city, state, and federal government. | Coastal Flooding |
| Muis et al.^e^ | Assessing flood risk under climate change and urban expansion for riverine and coastal flood hazard. | | Scenario-based analysis | | **Spatial Scale:**  Country-wide (Indonesia)  **Temporal Scale:**  Current and up to 2030 | - Projection of flood risk has demonstrated a dramatic increase in exposure of Indonesia for both fluvial and coastal flooding up to 2030.  - The main driver for increasing future flood risk in this country is urban expansion which put more exposed people and assets at flood risk.  - Significance of applying the mitigation strategies both in terms of urban planning strategies or structural measurements have been emphasized.  - Spatial planning has revealed to be more effective in case of riverine flooding. | Riverine and Coastal Flooding |
| Güneralp et al.^c^ | Evaluating changes in global pattern of flood hazard due to effect of exposure variation resulted by change in landscape. | | A coupled deterministic urban extent and hydrologic model | | **Spatial Scale:**  Global  **Temporal Scale:**  2000 - 2030 | - Coastal cities in Africa and Asia (developing countries) will have more impact on future flood loss due to urban growth than developed countries.  - Urban growth, even without considering the effect of climate change, will put more lives and assets at risk.  - To control the risk, proper adaptive mitigation strategies need to be placed.  - Planning and financing the infrastructure in cities which shows more growth in their extent and protect the future generation from the coastal and fluvial flooding hazard. | Coastal Flooding |
| Winsemius et al.^h^ | Evaluating the effect of socio-economic development along with the importance of climate change on future river flood risk. | | Scenario-based analysis | | **Spatial Scale:**  Global  **Temporal Scale:**  2010-2080 | - In the developing regions, the effect of socio-economic fabrics in increasing future flood risk was notable and can be intensified by the effect of climate change.  - Mitigation plans and adaptation strategies can significantly reduce the flood risk in future.  - The cost of the mitigation strategies is usually less than the benefits that can be gained by using them to alleviate flood risk. | Riverine Flooding |
| Ward et al.^g^ | Proposing a global framework for evaluating the cost and benefit of flood structural measurements. | | Scenario-based analysis | | **Spatial Scale:**  Global  **Temporal Scale:**  Current – 2100 | - Eliminating the effect of flood protection systems on calculating the current and future flood risk results in overestimation of the risk.  -Using Dyke as a structural mitigation measurement results in controlling the flood risk in many regions around the world but not everywhere.  - In some regions, a reasonable investment results in reducing the flood risk to lower level of current situation even though climate change and socioeconomic developments tend to increase the risk. | Riverine Flooding |

^a^Aerts et al. (2014). ^b^Bouwer et al. (2010). ^c^Güneralp et al. (2015). ^d^Jongman et al. (2012). ^e^Muis et al. (2015). ^f^Hallegatte, et al. (2013). ^g^Ward et al. (2017). ^h^Winsemius et al. (2016).
