# Supplementary material for: The Role of Urban Growth in Resilience of Communities Under Flood Risk
Source: Earths Future. 2020 Mar 20;8(3):e2019EF001382. doi: 10.1029/2019EF001382 (PMC7375139; doi:10.1029/2019EF001382)
Supplement: Supplementary file 4 — Table S3 [file EFT2-8-e2019EF001382-s004.docx]

Table 3
Summary of Studies Focusing on the Effect of Urban Growth on Policy Implementation towards a Resilient Community

| Authors | Prospective | Method | Scale of analysis | Results | Flooding type |
| --- | --- | --- | --- | --- | --- |
| Birkland et al.^a^ | Reviewing the flood mitigation policies.  Addressing some of the environmental concern of the current mitigation strategies and proposing some ways to reduce the flood risk without promoting catastrophic losses and environmental damage. | **-** | **-** | - Mitigation policies that is being adopted to control the flood losses currently focus on reducing the losses to property and people and they are not consider some ecological consequences associated with them.    - For applying a comprehensive plane, the role of federal government is undeniable. Since the funding from federal government is associated with flood risk management plans such as insurance and structural measurements, they need to continuously evaluate the effectiveness of their programs.  - Since the local government are the actual policy implementation, they need to effectively apply the funding as land use planning and measurements in order to save the community from the future flood hazard. | Riverine Flooding |
| Brody et al.^c^ | Identifying the consequences of flood intensity and urban development pattern on putting more property and lives at higher risk. | Regression Analysis | **Spatial Scale:**  144 coastal counties and communities along the Gulf of Mexico including: Florida, Georgia, Alabama, Mississippi, Louisiana, and Texas  **Temporal Scale:**  2001-2005 | - High intensity development pattern reduces losses and vice versa.  - The jurisdictions with more percentage of lands located in 100-year flood plain experience more losses in the flood events.  -Wetlands play an effective role in reducing flood losses. Using wetlands for construction will increase run off and increase flood damage.  - Increase in number of housing units as well as median household income increases flood losses. | Riverine and Coastal Flooding |
| Glavovic et al.^f^ | Emphasizing on the role of land-use planning on natural hazards | **-** | **Spatial Scale:**  New Zealand | - For applying land-use planning to mitigate the losses in hazard, an understanding of hazard, priorities in risk measures, and providing national guidance for the communities susceptible to the hazard are need. | Riverine and Coastal Flooding |
| Brody et al.^d^ | Evaluating the effect of planning and development decision on property damage in case of flooding events. | Regression Analysis | **Spatial Scale:**  383 non-hurricane flood events have been studied across 54 coastal counties in Florida  **Temporal Scale:**  1997 to 2001 | -Wetlands play an effective role on reducing flood loss and can be utilized as a natural mitigation plan.  - Dams do not significantly alleviate flood losses in Florida if planning strategies do not consider the influence of biophysical, socio-economic, and planning decision variables.  - Emphasizing the effectiveness of FEMA CRS program in reducing property damage resulting from floods. | Riverine and Coastal Flooding |
| Highfield et al.^g^ | Evaluating the effectiveness of local mitigation activities in reducing flood losses | Survey distribution and  Regression Analysis | **Spatial Scale:**  National wide study, USA  **Temporal Scale:**  1999–2009 | - In the areas where development in and around the floodplain has not already taken place, the results suggest that a nonstructural avoidance strategy of development should be pursued.  - Open space policies should be adopted through land acquisition, keeping public parcels vacant, or regulations that prohibit new buildings or filling on the land to decrease vulnerability of growing communities.  - As an overall approach, open space protection offers a policy vehicle for keeping structures out of the most vulnerable areas where they are most likely to incur damage while also conserving other beneficial services provided by the natural environment | Riverine and Coastal Flooding |
| Berke et al.^d^ | Investigating the effectiveness of disaster recovery and resiliency plan. | Survey distribution/  Multivariate Analysis | **Spatial Scale:**  Coastal counties in eight states along the Atlantic and Gulf coasts  between Virginia and Louisiana  **Temporal Scale:**  2007-2012 | - Planning for disaster recovery receives limited support within the study region.  - The recovery and resiliency plans are not sufficient.  - There is an essential need for more research on urban rebuilding and ultimately on community disaster resiliency and recovery. | Riverine and Coastal Flooding |
| Brody et al.^e^ | Examining the impact of land use/land cover characteristics on flood losses | Survey distribution and  Regression Analysis | **Spatial Scale:**  coastal watershed in southeast Texas, USA  **Temporal Scale:**  1999–2009 | - Specific types of surrounding LULCs impact observed flood losses.  - Some guidance have been provided in which neighborhoods can be developed more resiliently over the long term | Riverine and Coastal Flooding |
| Sadiq &  Noonan^h^ | Evaluating the characteristics of a community which result in a better performance in community Rating System strategy implemented by FEMA. | Regression  Analysis | **Spatial Scale:**  National-wide study  **Temporal Scale:**  1990- 2013 | - Communities behave differently in National wide mitigation programs such as CRS.  - More informed-communities and communities with lower property values, lower flood risk, and lower population densities respond better to CRS system.  - FEMA should invest more on evaluating the effectiveness of the CRS system and update some of the scoring system in order to make the communities more resilient to flooding hazard.  - FEMA should adopt some policies to encourage more communities and household to participate on flood mitigation measures to reduce the future losses. | Riverine and Coastal Flooding |

^a^Birkland et al. (2003), ^b^Berke et al. (2014), ^c^Brody et al. (2007), ^d^Brody et al. (2011), ^e^Brody et al. (2014), ^f^Glavovic et al. (2010), ^g^Highfeild et al. (2013), ^h^Sadiq & Noonan, S. (2015)
